# Does urbanization cause stress in wild birds during development? Insights from feather corticosterone levels in juvenile house sparrows (*Passer domesticus*)

**DOI:** 10.1002/ece3.4788

**Published:** 2018-12-21

**Authors:** Erika Beaugeard, François Brischoux, Pierre‐Yves Henry, Charline Parenteau, Colette Trouvé, Frédéric Angelier

**Affiliations:** ^1^ Centre d’Études Biologiques de Chizé (CEBC) UMR 7372 CNRS‐Université de La Rochelle Villiers‐en‐Bois France; ^2^ Centre de Recherches sur la Biologie des Populations d’Oiseaux (CRBPO) CESCO UMR 7204 Sorbonne Universités‐MNHN‐CNRS‐UPMC Paris France

**Keywords:** birds, CORT, development, morphology, stress, urbanization

## Abstract

Urban landscapes are associated with abiotic and biotic environmental changes that may result in potential stressors for wild vertebrates. Urban exploiters have physiological, morphological, and behavioral adaptations to live in cities. However, there is increasing evidence that urban exploiters themselves can suffer from urban conditions, especially during specific life‐history stages. We looked for a link between the degree of urbanization and the level of developmental stress in an urban exploiter (the house sparrow, *Passer domesticus*), which has recently been declining in multiple European cities (e.g., London, UK). Specifically, we conducted a large‐scale study and sampled juvenile sparrows in 11 urban and rural sites to evaluate their feather corticosterone (CORT) levels. We found that juvenile feather CORT levels were positively correlated with the degree of urbanization, supporting the idea that developing house sparrows may suffer from urban environmental conditions. However, we did not find any correlation between juvenile feather CORT levels and body size, mass, or body condition. This suggests either that the growth and condition of urban sparrows are not impacted by elevated developmental CORT levels, or that urban sparrows may compensate for developmental constraints once they have left the nest. Although feather CORT levels were not correlated with baseline CORT levels, we found that feather CORT levels were slightly and positively correlated with the CORT stress response in juveniles. This suggests that urban developmental conditions may potentially have long‐lasting effects on stress physiology and stress sensitivity in this urban exploiter.

## 1 INTRODUCTION

Urbanization induces profound and rapid landscape changes, which are associated with a reduction and alteration of natural habitats (Adams & Klobodu, 2017; Gil & Brumm, 2014; Marzluff, Bowman, & Donnelly, 2001; McKinney, 2002; Xu, Xie, Qi, Luo, & Wang, 2018). In cities, species have to cope with intense biotic and abiotic changes in their environment, such as urban features including buildings and roads (McKinney, 2002; Seress & Liker, 2015), different types or quantities of food (Haverland & Veech, 2017; Newsome et al., 2014; Seress & Liker, 2015; Vuorisalo et al., 2003), modifications to vegetation and the presence of exotic plants (Chace & Walsh, 2006; McKinney, 2006), and changes in the local climate due to anthropogenic activities (Seress & Liker, 2015). Urban species also have to face biochemical (Cai & Calisi, 2016; Gorissen, Snoeijs, Duyse, & Eens, 2005; Grimm et al., 2008; Seress & Liker, 2015), noise (Francis, Kleist, Ortega, & Cruz, 2012; Halfwerk et al., 2011; Meillère, Brischoux, & Angelier, 2015; Slabbekoorn et al., 2010), light (Kempenaers, Borgström, Loës, Schlicht, & Valcu, 2010; Navara & Nelson, 2007; Ouyang et al., 2017; Stone, Harris, & Jones, 2015), and/or microwave pollution (Balmori, 2009; Everaert & Bauwens, 2007). Considering these environmental modifications, the presence or absence of a given species in the urban landscape mainly depends on its ability to adjust to these novel environmental conditions (Candolin & Wong, 2012). Many species cannot cope with these changes (i.e., “urban avoiders”; Blair, 1996), and species richness decreases as the level of urbanization increases, leading to homogenized communities in cities (Gil & Brumm, 2014; McKinney, 2006). However, a few species can survive and reproduce in cities, and they may even benefit from urban conditions (i.e.*, *“urban exploiters”; Blair, 1996; Kark, Iwaniuk, Schalimtzek, & Banker, 2006).

Avian species are known to exhibit important changes in behavior, physiology, and morphology in urbanized areas (Gil & Brumm, 2014; Kark et al., 2006; Seress & Liker, 2015). For example, noise and light pollution are known to affect the reproductive behavior of small passerines like European robins (*Erithacus rubecula*), great tits (*Parus major*), pied flycatchers (*Ficedula hypoleuca*) and house sparrows (*Passer domesticus*; Miller, 2006; Halfwerk & Slabbekoorn, 2009; Kempenaers et al., 2010; de Jong et al., 2015; Meillère, Brischoux, & Angelier, 2015) and, consequently, their reproductive performance (Francis, Ortega, & Cruz, 2009; Halfwerk et al., 2011; Kempenaers et al., 2010; Kight & Swaddle, 2011; Kleist, Guralnick, Cruz, Lowry, & Francis, 2017). Similarly, an urban diet can have detrimental effects on reproductive performance (Demeyrier, Charmantier, Lambrechts, & Grégoire, 2017; Peach, Mallord, Ockendon, Orsman, & Haines, 2015; Plummer, Bearhop, Leech, Chamberlain, & Blount, 2013) and lead to nutritional stress in urban birds such as corvids (Heiss, Clark, & McGowan, 2009; Jones & Reynolds, 2008). These nutritional constraints can also affect development. For example, in great tits and house sparrows, urban individuals are also usually smaller and lighter than rural ones (Biard et al., 2017; Meillère et al., 2017; Meillère, Brischoux, Parenteau, & Angelier, 2015).

A small body size in urban individuals may be a result of developmental conditions. Corticosterone (CORT) is an important hormone that is relevant when evaluating the constraints that may occur during development. CORT is a glucocorticoid hormone that is secreted by the Hypothalamus–Pituitary–Adrenal axis (HPA) in response to unpredictable events and mediates allostasis in birds (i.e., stability through changes; McEwen & Wingfield, 2003; Landys, Ramenofsky, & Wingfield, 2006). High levels of CORT in birds are often associated with lower performance. In particular, high levels of CORT are associated with poor developmental conditions in chicks (Love, McGowan, & Sheriff, 2012; Wada, Salvante, Wagner, Williams, & Breuner, 2009), and a higher risk of reproductive failure in breeders (Angelier, Wingfield, Weimerskirch, & Chastel, 2010; Bonier, Martin, Moore, & Wingfield, 2009; Bonier, Moore, Martin, & Robertson, 2009; Cyr & Romero, 2007; Wingfield & Romero, 2001). Consequently, the study of CORT levels may be used to assess the ability of individuals and species to cope with urban conditions (Bonier, 2012; Zhang et al., 2011). For example, studies have shown that CORT secretion increases with nutritional stress in multiple species, such as white‐crowned sparrows (*Zonotrichia leucophrys*), eastern bluebirds (*Sialia sialis*), and snow petrels (*Pagodroma nivea*; Lynn, Breuner, & Wingfield, 2003; Lynn, Prince, & Phillips, 2010; Angelier, Wingfield, Parenteau, Pellé & Chastel, 2015). CORT levels are also correlated with other urban constraints, such as pollution, predation pressure, and/or human disturbance (Bonier, 2012; Cyr & Romero, 2007; Foltz et al., 2015; Love et al., 2012; Meillère et al., 2016; Ouyang et al., 2015; Zhang et al., 2011).

The house sparrow is commensal with humans and is considered to be one of the most highly adapted species to urban conditions (Anderson, 2006). Although this species was previously a widespread avian urban exploiter, urban populations of house sparrows have been strongly declining in European cities in the past few decades, particularly in highly urbanized cities like London (UK) and Antwerp (Crick, Robinson, Appleton, Clark, & Rickard, 2002; De Coster, Laet, Vangestel, Adriaensen, & Lens, 2015; Laet & Summers‐Smith, 2007; Shaw, Chamberlain, & Evans, 2008; Summers‐Smith, 2003). Recently, it has been suggested that urban conditions could be especially detrimental to developing sparrows: Urban conditions correlate negatively with growth, body size, and feather quality (Meillère et al., 2017; Meillère, Brischoux, Parenteau, et al., 2015; Seress et al., 2012). Previously, circulating blood CORT levels and body condition have been measured in adults and juveniles of both urban and rural populations, but no difference has been found (Bókony, Seress, Nagy, Lendvai, & Liker, 2012; Meillère et al., 2017; Meillère, Brischoux, Parenteau, et al., 2015). In a recent study, Hudin et al. (2018) compared feather CORT levels between urban and rural sparrows, and they did not find any significant difference in feather CORT levels between these populations. However, they focused on a specific geographical area and considered urbanization as a categorical factor without precisely quantifying the degree of urbanization (see Liker, Papp, Bókony, & Lendvai, 2008). Such a quantitative approach would be beneficial to generalize these previously reported results (Hudin et al., 2018) and to test whether the effect of urbanization on CORT levels may vary according to the degree of urbanization.

In this study, we investigated the impact of urbanization on the stress physiology of growing house sparrows. It is difficult to measure CORT levels in house sparrow nestlings because plasma CORT levels vary throughout the developmental period (Wada et al., 2008, 2009), and nests are very difficult to access in cities. To solve these problems, we used feather samples. House sparrow nestlings retain the feathers that they grow during development for several weeks after fledging, so it is possible to sample feathers after juveniles leave the nest. Further, circulating CORT is incorporated into the feather matrix during feather growth (Bortolotti, Blas, & German, 2008; Jenni‐Eiermann et al., 2015) and, therefore, feathers that were grown in the nest provide an integrative measure of developmental CORT levels. However, it is still important to measure circulating plasma CORT levels, because they can help us understand the long‐term effects of a nestling's exposure to stress. Plasma CORT levels give information about juvenile condition by measuring the normal expression of CORT (baseline CORT level), and the functionality of the HPA axis later in life by measuring their response to stress (stress‐induced CORT level). Using this method, we investigated (a) the link between urbanization and feather CORT levels in juvenile sparrows, (b) the link between feather CORT levels and morphological attributes of juveniles (body size, mass, and condition), and (c) the link between feather CORT levels and plasma CORT levels. To do so, we led a large‐scale sampling effort (feather plucking and morphological measurements of juvenile sparrows) thanks to a network of volunteers and qualified ornithologists who sampled 11 sites located through an urbanization gradient (from rural areas to large cities). First, we predicted that if urbanization is associated with important developmental constraints, feather CORT levels would increase as the degree of urbanization increased. Second, if developmental stress affects both CORT regulation and growth, we predicted that body size and condition would correlate negatively with feather CORT levels in juvenile sparrows. Finally, if developmental stress affects the HPA axis with long‐lasting effects on CORT regulation, we expected that feather CORT levels may be positively correlated with plasma CORT levels in juveniles. Alternatively, feather CORT levels and juvenile plasma CORT levels may be independent if such long‐lasting effects are not apparent.

## 2 MATERIALS AND METHODS

### 2.1 Ethics statement

This study was carried out in accordance with all applicable institutional and/or national guidelines for the care and use of animals. All experimental procedures were approved by the “Comité d'Ethique en Expérimentation Animale Poitou‐Charentes”, France (authorization number: CE2012–7). Permits for the capture, sampling and banding of house sparrows were issued by the “Centre de Recherches sur la Biologie des Populations d'Oiseaux” (CRBPO) to all the ringers involved in the sampling.

### 2.2 Study sites and captures

**Figure 1:**
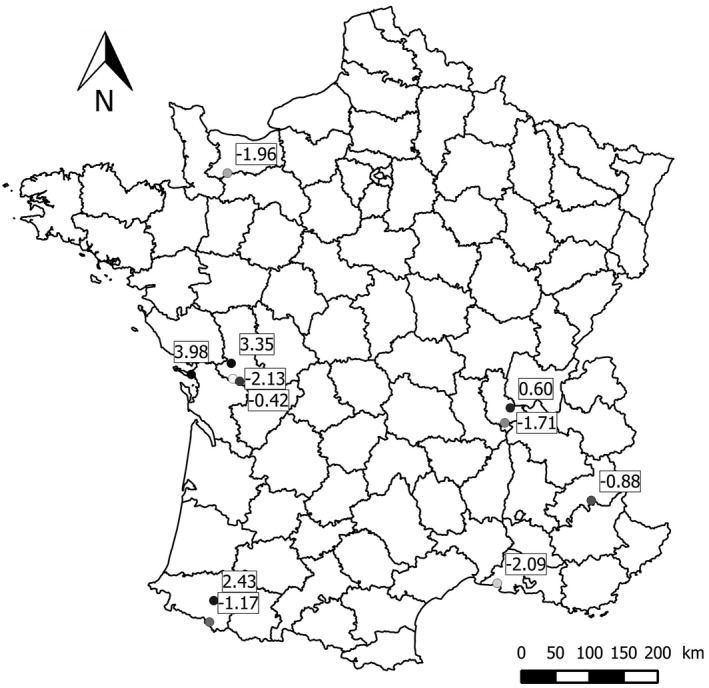
Geographical localization of the 11 capture sites sampled in this study

In 2013 (June–August), a total of 111 juvenile house sparrows were captured with mist nets at 11 sites in France (Figure 1; geographic coordinates of the capture sites and sample sizes for each population are summarized in Table 1). The 11 sites differed in urbanization level, ranging from sparsely populated areas (e.g., isolated farms, small villages) to highly urbanized city centers. To quantify the degree of urbanization of each capture site, we used the method developed by Liker et al. (2008) for house sparrows (see also Meillère, Brischoux, Parenteau, et al., 2015; Meillère et al., 2017). Briefly, we used digital aerial photographs of 1 km^2^ areas around each capture site that we divided into 100 cells. For each capture site, we extracted five habitat characteristics as follows: mean building density score, number of cells with high building density, mean vegetation density score, number of cells with high vegetation density, and number of cells with roads. Then, we used the PC1 value from a principal component analysis (PCA) of these five variables to attribute an urbanization score to each site (Table 1). The PC1 accounted for 93.3% of the total variance and was strongly positively correlated with artificial surfaces (building density and roads; all *r* > 0.806) and negatively with vegetation cover (all *r* < −0.980).

**Table 1:** Habitat characteristics of the capture sites and corresponding sample sizes. Sites are ordered from the most to the least urbanized site (in bold: PC1 values from a principal component analysis conducted on the five habitat variables). Sites with * are those used for additional morphological and physiological measurements. The sites are shown in the map in Figure 1

Capture site (geographic coordinates)	Habitat characteristics						Sample sizes
	Mean building density score	Number of cells with high building density	Mean vegetation density	Number of cells with high vegetation density	Number of cells with road	Urbanization score (PC1)	Juveniles
La Rochelle* (46.148; −1.154)	1.22	36	0.62	11	95	**3.98**	13
Niort* (46.313; −0.479)	1.18	24	0.82	11	97	**3.35**	10
Oloron‐Ste‐Marie (43.196; −0.611)	1.11	31	1.17	32	68	**2.43**	11
Lyon (45.777; 4.854)	0.52	7	1.52	57	89	**0.60**	7
Villefollet* (46.127; −0.268)	0.45	11	1.72	74	48	**−0.42**	8
Chorges (44.546; 6.266)	0.30	8	1.71	81	42	**−0.88**	10
Etsaut (42.913; −0.572)	0.34	3	1.92	92	54	**−1.17**	3
Givors (45.560; 4.772)	0.17	0	2.00	100	50	**−1.71**	15
Rully (48.824; −0.715)	0.15	2	1.97	97	27	**−1.96**	8
Saintes‐Maries‐de‐la‐mer (43.490; 4.401)	0.12	0	2.00	100	30	**−2.09**	13
Villiers‐en‐Bois* (46.147; −0.426)	0.11	1	1.98	98	23	**−2.13**	13

### 2.3 Feather collection (11 sites), morphological measurements, and blood sampling (4 sites)

Feathers were collected from all study sites. The two innermost rectrices of each individual were collected and stored in dry paper envelopes until laboratory analyses. We weighed (high‐resolution balance: ±0.01 mg) and measured the length (digital caliper: ±0.01 mm; see Meillère et al., 2017) of all feathers. In addition to feather collections, morphological measurements and blood samples were collected at 4 of the 11 sites (two urban and two rural; see Table 1). For all juveniles captured at these four sites, body mass (electronic balance: ±0.1 g), wing length (steel rule: ±1 mm), tarsus length, and bill length (caliper: ±0.1 mm) were measured. Additionally, fat and muscle scores were recorded as detailed in Kaiser (1993), Brown (1996) and Leloutre, Gouzerh, and Angelier (2014). For consistency and to avoid potential methodological bias, morphological measurements were all collected by the same person. In addition to morphological measurements, juveniles from these four sites were bled within 3 min of capture (mean ± *SE*: 2 min 39 s ± 2 s; range: 1 min 13 s – 3 min 45 s) to quantify baseline CORT concentration (Romero & Reed, 2005). Following this first blood sample, birds were kept in cloth bags and sampled a second time 30 min after capture to obtain stress‐induced CORT levels (Wingfield, Davey, Peter, & Tobe, 1994). CORT levels are at a maximum after 30‐min of restraint in this species (Romero, Cyr, & Romero, 2006), and therefore, this allowed us to assess the CORT stress response (Wingfield et al., 1998). We defined the “increase in plasma CORT” as the difference between stress‐induced CORT and baseline CORT levels. All blood samples were collected from the alar vein using a 25‐gauge needle and heparinized microcapillary tubes (up to 150 µl for CORT assay). Blood samples were centrifuged (4,500 rpm, 7 min), and plasma and red blood cells were separated (plasma for CORT assay and red blood cells for molecular sexing). Then, they were kept at −20°C until laboratory analyses at the “Centre d'Etudes Biologiques de Chizé” (hereafter CEBC).

To assess juvenile body condition, we used the “scaled mass index” (SMI) as recommended by Peig & Green (2009, 2010). The SMI adjusts the mass of all individuals to that which would be expected if they all had the same body size (Peig & Green, 2009). We used tarsus length to calculate the SMI because it had the greatest correlation with body mass (tarsus length: *r* = 0.588, *p* < 0.001; wing length: *r* = 0.313, *p* = 0.041). The SMI was computed for each individual as follows:

$${\text{SMI}}_{i}=M_{i}\times {\left(L_{0}/L_{i}\right)}^{b}$$

where *M*_i_ and *L*_i_ are body mass and tarsus length of the individual i, respectively, *L*_0_ is the arithmetic mean value of tarsus length for the whole study population (*L*_0_ = 18.18 mm, *n* = 43), and *b* is the slope estimate of a standardized major axis (SMA) regression of log‐transformed body mass on log‐transformed tarsus length (*b* = 1.86).

### 2.4 Molecular sexing and CORT analyses

The sex of juveniles was determined by molecular sexing as detailed in Fridolfsson and Ellegren (1999). DNA was extracted from blood samples, and PCR reactions were performed in order to amplify genomic DNA. DNA sequences with only the CHDIZ fragment correspond to males, whereas DNA sequences with the CHDIZ and/or the CHDIW fragment correspond to females. Plasma concentrations of CORT were measured in duplicate by radio‐immunoassay, as previously described (Lormée, Jouventin, Trouve, & Chastel, 2003). Feather CORT assays were conducted at the CEBC by following previously validated methods (Bortolotti et al., 2008) with minor improvements (see Meillère et al., 2016). Specifically, 10 ml of methanol (HPLC grade) were added to each feather sample to extract CORT from the whole feather. Then, feathers were incubated at 50°C overnight in a shaking water bath, the methanol was separated from feather material, and the feather remnants were washed before being added to the original methanol extract. Then, feather extracts were analyzed by radio‐immunoassay at the CEBC as previously described (Lormée et al., 2003). Feather CORT levels were expressed either as ng of CORT per mg of feather (ng/mg) or as ng of CORT per mm of feather (ng/mm). The minimum detectable CORT level was 0.83 ng/ml. All samples were run in three assays, and the intra‐ and inter‐assay coefficients of variation were 7.07% and 9.99%, respectively.

### 2.5 Statistical analyses

All statistical analyses were performed in R 3.4.3 (R Core Team, 2016). First, to test the correlation of urbanization with feather CORT levels in juveniles, we fitted linear mixed models (LMMs, normal error distribution, identity link function) with “capture site” (a total of 11 sites) as a random factor to control for the non‐independence of individuals captured at the same site. We used “urbanization” (PC1 score) and “capture date” as fixed effects in our model. We did not include the “sex” in this analysis because it was unknown for all individuals that were not bled (60 birds from 7 sites). Second, to test whether developmental stress affects both CORT regulation and growth, we fitted LMMs (normal error distribution, identity link function) with “SMI,” “tarsus length,” “body mass,” “baseline CORT,” “stress‐induced CORT levels,” or “increase in plasma CORT” as response variables. We used “capture site” as a random factor, and “feather CORT level” and “sex” as fixed effects in these models. We verified that all models met the assumptions of equal variances and normal residuals. Baseline CORT levels were log_10_‐transformed to ensure the normality of model residuals, but we present non‐transformed values to facilitate interpretation. Feather CORT levels in ng/mm were highly correlated with feather CORT levels in ng/mg (*r* = 0.837; *p* < 0.001), and all the results were qualitatively similar when using one measure or the other. Therefore, we only present the feather CORT data expressed in ng/mg in the rest of the manuscript (but see Supporting Information Table S1 and Figure S1 for all results and graph with feather CORT expressed in ng/mm).

## 3 RESULTS

### 3.1 Urbanization and feather CORT levels (11 sites)

**Figure 2:**
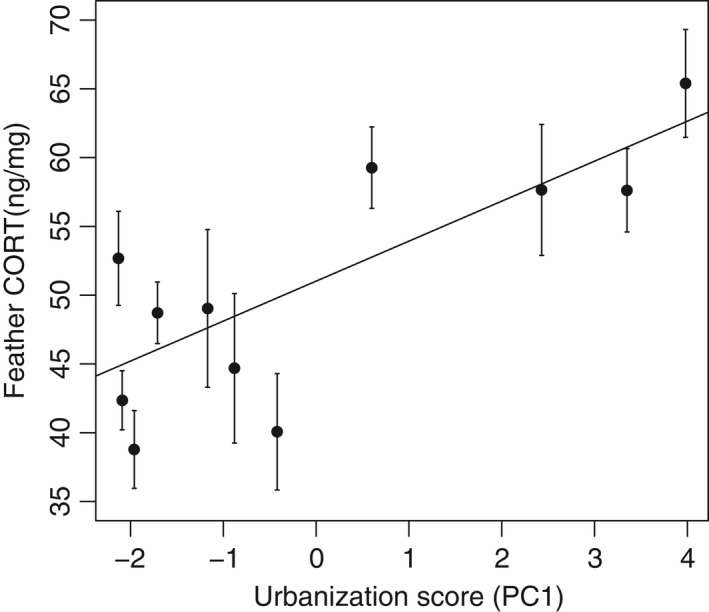
Relationship between the degree of urbanization and feather CORT level expressed in ng/mg in juvenile sparrows. The line represents a significant relationship between feather CORT level and urbanization score

Feather CORT levels were significantly and positively correlated with urbanization score (LMM: *t* = 3.79, *p* = 0.004; Figure 2). Feather CORT levels were in mean 19.45% higher in the most urban site relative to the most rural site. Feather CORT levels were not explained by the capture date (*t *= −0.03, *p* = 0.974).

### 3.2 Relationship between feather CORT levels and body size and condition (4 sites)

Neither SMI, tarsus length, nor body mass were explained by feather CORT levels, sex, or the “CORT_f_ x Sex” interaction (Table 2a–c).

**Table 2:** Results of the linear mixed models (LMMs) when investigating the influence of feather CORT levels on body size, body condition, and plasma CORT levels, with feather CORT expressed in ng/mg. All models include capture site as a random factor. Estimates are for males in comparison to females

Model	Parameter	Estimate ±SE	*t*	*p*	95% CI
(a) SMI	Intercept	25.49 ± 1.40	18.18	<0.001	22.64; 28.34
	CORTf	−0.01 ± 0.02	−0.57	0.571	−0.06; 0.03
	Sex	−1.96 ± 1.98	−0.99	0.330	−5.98; 2.07
	CORTf x Sex	0.03 ± 0.03	0.98	0.332	−0.04; 0.10
(b) Tarsus length	Intercept	18.21 ± 0.55	33.16	<0.001	17.15; 19.27
	CORTf	−0.004 ± 0.009	−0.48	0.637	−0.02; 0.01
	Sex	1.13 ± 0.78	1.44	0.159	−0.38; 2.64
	CORTf x Sex	−0.01 ± 0.01	−1.006	0.322	−0.04; 0.01
(c) Body mass	Intercept	25.56 ± 1.46	17.54	<0.001	22.60; 28.52
	CORTf	−0.02 ± 0.02	−1.02	0.317	−0.07; 0.02
	Sex	0.88 ± 2.08	0.42	0.675	−3.35; 5.11
	CORTf x Sex	0.0003 ± 0.04	0.009	0.993	−0.07; 0.07
(d) Baseline CORT level	Intercept	1.30 ± 1.25	1.05	0.305	1.10; 3.71
	CORTf	−0.02 ± 0.02	−0.81	0.428	−0.06; 0.02
	Sex	−1.57 ± 1.91	−0.82	0.419	−5.26; 2.12
	CORTf x Sex	0.01 ± 0.03	0.30	0.764	−0.05 0.07
(e) Stress‐induced CORT level	Intercept	12.59 ± 5.61	2.24	0.031	1.18; 24.01
	CORTf	0.17 ± 0.09	1.87	0.070	−0.01; 0.35
	Sex	5.77 ± 7.65	0.75	0.456	−9.79; 21.33
	CORTf x Sex	−0.20 ± 0.13	−1.52	0.138	−0.47; 0.07
(f) Increase in plasma CORT	Intercept	9.72 ± 5.33	1.82	0.080	−1.24; 20.68
	CORTf	0.18 ± 0.08	2.18	**0.039**	0.01; 0.36
	Sex	7.25 ± 7.28	1.00	0.329	−7.71; 22.21
	CORTf x Sex	−0.21 ± 0.13	−1.63	0.115	−0.48; 0.05

### 3.3 Relationship between feather CORT level and plasma CORT levels (4 sites)

**Figure 3:**
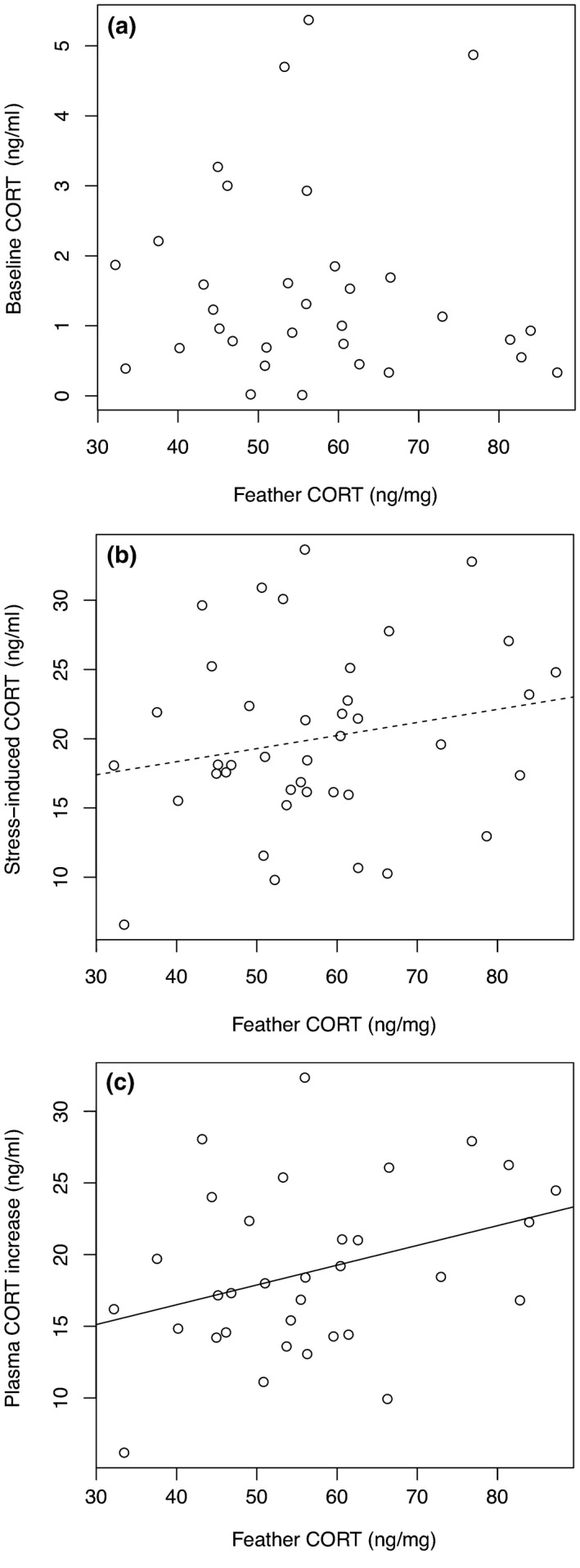
Relationship between feather CORT levels expressed in ng/mg and (a) baseline CORT level, (b) stress‐induced CORT level, and (c) increase in plasma CORT in juvenile sparrows. The dotted line represents a marginal and non‐significant relationship (*p* < 0.10); the solid line represents a significant relationship (*p* < 0.05)

Neither baseline CORT levels nor stress‐induced CORT levels were explained by feather CORT levels (Figure 3a,b), sex, or the “CORT_f_ x Sex” interaction (Table 2d,e), although stress‐induced CORT levels were positively but weakly associated with feather CORT levels (Table 2e). However, the increase in plasma CORT in juveniles was significantly and positively related to feather CORT levels (Table 2f; Figure 3c). Moreover, this increase was not significantly affected by sex or the “CORT_f_ x Sex” interaction, suggesting the effect of feather CORT levels on the increase in plasma CORT did not significantly differ between males and females (Table 2f).

## 4 DISCUSSION

The house sparrow may represent a sentinel species of the urban environment and, thus, is a good model to study the functional constraints of living in cities for urban exploiters. To better understand the potential causes of the recent decline of urban house sparrow populations, we conducted a large‐scale study to evaluate the impact of urbanization on the stress physiology of juvenile house sparrows. By using an integrative measure of CORT from feathers sampled across a rural–urban gradient, we found that urban juveniles had higher feather CORT levels than rural ones. These feathers grew during the developmental phase (i.e., in the nest); thus, our study highlights the functional constraints imposed by a city lifestyle and suggests that these constraints are most likely to occur during the developmental period prior to fledging. Feather CORT levels were not correlated to any juvenile attribute (body size, mass, or condition) except for the CORT stress response (the increase in CORT levels in response to a standardized stress protocol), suggesting that urban environmental conditions may affect the ontogeny of the CORT stress response in house sparrow nestlings.

### 4.1 Impact of urbanization on feather CORT level

We showed that an increasing level of urbanization was associated with higher feather CORT levels in juvenile house sparrows. This result partly contrasts with those from Hudin et al. (2018). In their study, they found no difference in feather CORT levels between urban and rural juvenile sparrows. Contrary to our study, they captured birds in early fall, when the post‐juvenile molt has already occurred in house sparrows. This could explain the discrepancy because most urban‐related constraints seem to occur during the developmental period when sparrows are growing their feathers in the nest (Meillère et al., 2017; Seress et al., 2012). Elevated feather CORT levels usually result from elevated circulating plasma CORT levels at the time of feather growth (Bortolotti et al., 2008), as experimentally demonstrated in pigeons (*Columbia livia domestica*) and European starlings (*Sturnus vulgaris*; Lattin, Reed, DesRochers, & Romero, 2011; Jenni‐Eiermann et al., 2015). Consequently, elevated feather CORT levels have been associated with challenging environmental conditions in various species (e.g., geese and eiders in Legagneux et al., 2013; tree and barn swallows (*Tachycineta bicolor*, *Hirundo rustica*) in Fairhurst et al., 2015, 2011; rhinoceros auklet (*Cerorhinca monocerata*) in Will et al., 2015; mallard duckling (*Anas platyrhynchos*) in Johns, Marchant, Fairhurst, Speakman, & Clark, 2017) and are associated with reduced survival in house sparrows, specifically (e.g., Koren et al., 2012). Our results support the hypothesis that urban sparrows are constrained during their development and suggest that urban juvenile sparrows may have a lower survival probability than rural ones (Meillère et al., 2017; Meillère, Brischoux, Parenteau, et al., 2015; Seress et al., 2012). This interpretation is supported by multiple ecological studies, which have reported a negative impact of urbanization on house sparrow populations (e.g., population decline: De Coster et al., 2015; reduced breeding success: Seress et al., 2012; reduced body size and feather quality: Meillère et al., 2017). However, exposure to moderate CORT levels during development can also be beneficial under some circumstances (reviewed in Madliger & Love, 2014). For example, Grava et al. (2013) found that elevated juvenile feather CORT levels were associated with potential benefits later in life (i.e., post‐fledging habitat quality, syrinx mass). Future studies should examine the fitness consequences of slight inter‐individual variation in feather CORT levels in juvenile sparrows.

Interestingly, visual inspection of our data suggests that urbanization may only be a constraint when it reaches a specific threshold (see. Figure 2). However, we lack statistical power to run non‐linear models (e.g., broken‐stick models), and sampling additional populations would be required to properly test this hypothesis.

There are numerous potential sources of stress for birds developing in an urban environment, which could impact nestlings either directly or indirectly through the modification of environmental constraints (Bonier, 2012; Chace & Walsh, 2006; Meillère, Brischoux, & Angelier, 2015; Seress & Liker, 2015). Elevated feather CORT levels could result from a change in diet and food quality in urban environments. Recently, Hudin et al. (2018) experimentally showed that an urban diet is associated with elevated feather CORT levels in juvenile house sparrows, supporting the idea that the urban diet may not be suitable for developing house sparrow nestlings (Meillère et al., 2017). Indeed, nestling house sparrows need a protein‐rich diet to grow properly (Anderson, 2006; White, 2008), but the availability of invertebrates is lower in cities relative to rural areas (Chace & Walsh, 2006; Hudin et al., 2017; Moudrá, Zasadil, Moudrý, & Šálek, 2018; Paker, Yom‐Tov, Alon‐Mozes, & Barnea, 2014; Summers‐Smith, 2003), and urban house sparrow parents feed their chicks a lower quality diet than their rural counterparts (Seress & Liker, 2015). In addition, urban food may contain heavy metals, which may contaminate nestlings (Dauwe, Janssens, Bervoets, Blust, & Eens, 2004; Raupp, Shrewsbury, & Herms, 2010; Zvereva & Kozlov, 2010) and lead to potential effects on growth, behavior or the immune system (e.g., pied flycatchers in Eeva, Hasselquist, Tummeleht, Nikinmaa, & Ilmonen, 2005; great tits in Gorissen et al., 2005; feral pigeons (*Columbia livia*) in Chatelain, Gasparini, & Frantz, 2016; reviewed in Montiglio & Royauté, 2014). A low‐quality and/or contaminated diet could be associated with nutritional stress and, therefore, with elevated feather CORT levels in urban chicks (e.g., food restriction in rhinoceros auklets: Will et al., 2014, 2015; heavy metal contamination in blackbirds (*Turdus merula)*: Meillère et al., 2016), especially in house sparrows (Hudin et al., 2018). Feather CORT levels can also be affected by weather conditions, as previously shown in several species (Legagneux et al., 2013), including the house sparrow (Treen, Hobson, Marchant, & Bortolotti, 2015). Unfortunately, climate data were not available for most sites, so we could not test this hypothesis. However, our results are unlikely to be biased by inter‐site variation in climate because the rural and urban sites were not spatially segregated (see Figure 1).

Higher feather CORT levels in urban nestlings could also be a result of environmental perturbations, such as noise and light pollution. Noise pollution could indirectly affect nestlings by disrupting aspects of parental care such as incubation commitment or brood provisioning (Injaian, Taff, & Patricelli, 2018; Meillère, Brischoux, & Angelier, 2015; Schroeder, Nakagawa, Cleasby, & Burke, 2012). Noise pollution could also directly disturb the nestlings, and as a result, increase nestling CORT secretion as previously shown in a few bird species (Kleist et al., 2017; Davies, Beck, & Sewall, 2018; but see Angelier, Meillère, Grace, Trouvé, & Brischoux, 2016). Previous studies have demonstrated that urban noise is associated with changes in nestlings growth, metabolism, and stress response, and with a reduced probability of survival in several bird species (e.g., tree swallows in Leonard & Horn, 2008; Injaian et al., 2018; white‐crowned sparrows (*Zonotrichia leucophrys orienta*) in Crino, Johnson, Blickley, Patricelli, & Breuner, 2013; house sparrows in Meillère, Brischoux, Ribout, & Angelier, 2015; Brischoux, Meillère, Dupoué, Lourdais, & Angelier, 2017; western bluebirds (*Sialia mexicana*) in Kleist et al., 2017). Light pollution may also result in an increase in feather CORT because it artificially increases the amount of time nestlings are active. For instance, an experimental study on great tits showed that urban light was associated with increased brood provisioning (Titulaer, Spoelstra, Lange, & Visser, 2012) and, therefore, with increased begging activity in urban nestlings (Ouyang et al., 2015; Titulaer et al., 2012). Increased activity (especially begging activity) has been associated with increased CORT levels in wild birds (Kitaysky, Kitaiskaia, Piatt, & Wingfield, 2003; Loiseau, Sorci, Dano, & Chastel, 2008; reviewed in Landys et al., 2006). Since high‐quality food may be limited in cities (Seress & Liker, 2015), urban nestlings may be expending more energy by begging than they are receiving, leading to nutritional stress and increased feather CORT levels (Will et al., 2014).

### 4.2 Feather CORT levels and body size

In previous studies, we found that body size decreases as the degree of urbanization increases in house sparrows (Meillère et al., 2017; Meillère, Brischoux, Parenteau, et al., 2015). Although we found a positive correlation between feather CORT levels and the degree of urbanization, we surprisingly did not find any significant relationship between feather CORT levels and body size in juvenile house sparrows. As feather and structural growth occur simultaneously in the nest, our results suggest that CORT exposure during the post‐hatching period is not directly correlated with nestling growth. Previous studies have shown that elevated plasma and feather CORT levels can be correlated with reduced body size and mass in pigeon and sparrow nestlings (Grace, Froud, Meillère, & Angelier, 2017; Jenni‐Eiermann et al., 2015). Interestingly, this correlation between CORT levels, body size, and body mass seems to attenuate and even disappear as the nestlings develop, suggesting that nestlings can compensate for a slow initial growth despite stressful or challenging conditions (Grace et al., 2017; Jenni‐Eiermann et al., 2015). This may explain why feather CORT levels and juvenile body size were not correlated in our study, although this may also result from a lack of statistical power (tarsus length was only available from 4 sites). Furthermore, CORT is progressively and irreversibly incorporated into feathers during growth and, therefore, feather CORT levels represent a sum of CORT exposure during feather growth (Bortolotti et al., 2008; Jenni‐Eiermann et al., 2015; Romero & Fairhurst, 2016; Will et al., 2014). Growth and juvenile body size may be affected by acute and temporary stressful events during growth, which are not identifiable in the integrated feather CORT measurement.

### 4.3 Feather CORT level and juvenile body condition and CORT levels

Juvenile body mass and condition were not influenced by feather CORT levels. Unlike body size, body mass and condition are labile, and thus are likely to be more dependent on individual characteristics and the local environmental conditions that are present closer to the time of capture (e.g., food, weather, predation; Krebs & Singleton, 1993; Gosler, 1996; Schulte‐Hostedde, Millar, & Hickling, 2001; Peig & Green, 2009). In contrast, feather CORT is determined weeks prior to the time of capture. This may blur a potential relationship between feather CORT levels and condition in juvenile sparrows. An alternative explanation may be that there is a progressive selective disappearance of individuals with low body condition in the population. Post‐fledging mortality is high in small passerines including house sparrows (Leloutre et al., 2014). In addition, higher feather CORT concentrations are associated with lower survival probability in adult house sparrows (Koren et al., 2012). This selection may eliminate individuals with a lower body mass and condition (see Lendvai, Loiseau, Sorci, & Chastel, 2009) and may, therefore, mask a potential relationship between developmental conditions (e.g., feather CORT levels) and juvenile attributes (body size, body mass and condition, and baseline CORT levels).

We measured baseline CORT and stress‐induced CORT in order to test whether the HPA axis and juvenile sensitivity to stress may be affected by developmental conditions and CORT exposure during development (i.e., feather CORT). We did not find evidence for any correlation between feather CORT levels and baseline CORT levels. However, we found a non‐significant positive trend between stress‐induced CORT levels and feather CORT levels. We also found a significant positive relationship between feather CORT levels and the CORT stress response (i.e., the increase in CORT levels in response to a standardized stressor) in juvenile sparrows. Such result has previously been found with a positive relationship between stress‐induced CORT levels and feather CORT levels in partridges (*Alectoris rufa*; Bortolotti et al., 2008). These results suggest that the CORT stress response at the juvenile stage may be associated with developmental CORT exposure. Several laboratory studies have previously reported an effect of developmental CORT exposure on the CORT stress response of individuals later in life (e.g., Spencer, Buchanan, Goldsmith, & Catchpole, 2003; Spencer, Evans, & Monaghan, 2009; Haussmann, Longenecker, Marchetto, Juliano, & Bowden, 2011; Marasco, Robinson, Herzyk, & Spencer, 2012; Crino, Driscoll, & Breuner, 2014). For instance, Spencer et al. (2009) showed that CORT‐fed zebra finch nestlings had a steeper increase in plasma CORT levels in response to an acute stress as juveniles. Stressful conditions during development (e.g., nutritional restrictions) without any manipulation of plasma CORT levels can also increase plasma CORT levels of juveniles in response to stress, as shown in red‐legged kittiwakes and western scrub‐jays (Kitaysky, Kitaiskaia, Wingfield, & Piatt, 2001; Pravosudov & Kitaysky, 2006). Thus, developmental stress or developmental CORT exposure may have long‐lasting effects on the CORT stress response of house sparrows. This suggests that urban developmental conditions may modify the functioning of the HPA axis in urban birds. It is possible that an intense CORT stress response may provide some benefits to urban individuals during the juvenile or the adult stage (e.g., improved survival, Angelier et al., 2010). To test this hypothesis, future studies would need to experimentally investigate the impact of developmental CORT exposure on the ontogeny of the HPA axis and the ability of individuals to subsequently cope with their environment.

## CONFLICT OF INTEREST

The authors have no conflict of interest to declare.

## AUTHOR CONTRIBUTIONS

EB, FB, PYH, and FA designed the study, conducted the statistical analyses, and drafted the manuscript; CP and CT assayed CORT levels in feather and blood. All authors gave final approval for publication.

## DATA ACCESSIBILITY

Raw data are available on Dryad, https://doi.org/10.5061/dryad.r617g73.
