# Supplementary material for: Does urbanization cause stress in wild birds during development? Insights from feather corticosterone levels in juvenile house sparrows (Passer domesticus)
Source: Ecol Evol. 2018 Dec 21;9(1):640–52. doi: 10.1002/ece3.4788 (PMC6342122; doi:10.1002/ece3.4788)
Supplement: Supplementary file 2 [file ECE3-9-640-s002.docx]

**Table S1:** Results of the linear mixed models (LMMs) when investigating the influence of feather CORT levels on body size, body condition, and plasma CORT levels, with feather CORT expressed in ng/mm. All models include capture site as a random factor. Estimates are for males in comparison to females.

| **Model** | **Parameter** | **Estimate ± SE** | ***t*** | ***p*** | **95% CI** |
| --- | --- | --- | --- | --- | --- |
| A) SMI | Intercept | 25.68 ± 1.64 | 15.66 | <0.001 | 22.35; 29.01 |
|  | CORTf | -0.18 ± 0.30 | -0.60 | 0.551 | -0.79; 0.43 |
|  | Sex | -2.00 ± 2.11 | -0.95 | 0.349 | -6.29; 2.29 |
|  | CORTf x Sex | 0.37 ± 0.39 | 0.94 | 0.353 | -0.42; 1.15 |
| B) Tarsus length | Intercept | 18.06 ± 0.66 | 27.28 | <0.001 | 16.78; 19.34 |
|  | CORTf | -0.02 ± 0.12 | -0.16 | 0.871 | -0.25; 0.21 |
|  | Sex | 1.05 ± 0.86 | 1.22 | 0.230 | -0.61; 2.70 |
|  | CORTf x Sex | -0.12 ± 0.16 | -0.78 | 0.439 | -0.43; 0.18 |
| C) Body mass | Intercept | 25.41 ± 1.75 | 14.55 | <0.001 | 22.04; 28.78 |
|  | CORTf | -0.24 ± 0.32 | -0.75 | 0.456 | -0.85; 0.37 |
|  | Sex | 0.63 ± 2.26 | 0.28 | 0.783 | -3.74; 4.99 |
|  | CORTf x Sex | 0.0065 ± 0.42 | 0.16 | 0.876 | -0.74; 0.87 |
| D) Baseline CORT level | Intercept | 1.71 ± 1.47 | 1.16 | 0.256 | -1.31; 4.73 |
|  | CORTf | -0.25 ± 0.26 | -1.96 | 0.347 | -0.80; 0.29 |
|  | Sex | -2.72 ± 2.02 | -1.34 | 0.191 | -6.88; 1.44 |
|  | CORTf x Sex | 0.34 ± 0.38 | 1.89 | 0.382 | -0.44; 1.11 |
| E) Stress-induced CORT level | Intercept | 15.94 ± 6.37 | 2.50 | 0.018 | 2.97; 28.90 |
|  | CORTf | 1.16 ± 1.12 | 1.04 | 0.306 | -1.11;3.44 |
|  | Sex | 4.62 ± 8.14 | 0.57 | 0.574 | -11.94; 21.19 |
|  | CORTf x Sex | -1.92 ± 1.49 | -1.29 | 0.207 | -4.95; 1.12 |
| F) Increase in plasma CORT | Intercept | 11.47 ± 6.02 | 1.90 | 0.068 | -0.91; 23.85 |
|  | CORTf | 1.64 ± 1.05 | 1.57 | 0.129 | -0.51; 3.79 |
|  | Sex | 7.92 ± 7.75 | 1.02 | 0.317 | -8.02; 23.85 |
|  | CORTf x Sex | -2.41 ± 1.46 | -1.65 | 0.112 | -5.41; 0.60 |
